# Exploring Deep Learning for Complex Trait Genomic Prediction in Polyploid Outcrossing Species

**DOI:** 10.3389/fpls.2020.00025

**Published:** 2020-02-06

**Authors:** Laura M. Zingaretti, Salvador Alejandro Gezan, Luis Felipe V. Ferrão, Luis F. Osorio, Amparo Monfort, Patricio R. Muñoz, Vance M. Whitaker, Miguel Pérez-Enciso

**Affiliations:** ^1^Centre for Research in Agricultural Genomics (CRAG) CSIC-IRTA-UAB-UB, Campus UAB, Barcelona, Spain; ^2^School of Forest Resources and Conservation, University of Florida, Gainesville, FL, United States; ^3^Blueberry Breeding and Genomics Lab, Horticultural Sciences Department, University of Florida, Gainesville, FL, United States; ^4^IFAS Gulf Coast Research and Education Center, University of Florida, Wimauma, FL, United States; ^5^Institut de Recerca i Tecnologia Agroalimentàries (IRTA), Barcelona, Spain; ^6^ICREA, Passeig de Lluís Companys 23, Barcelona, Spain

**Keywords:** genomic prediction, genomic selection, polyploid species, deep learning, epistasis, complex traits, strawberry, blueberry

## Abstract

Genomic prediction (GP) is the procedure whereby the genetic merits of untested candidates are predicted using genome wide marker information. Although numerous examples of GP exist in plants and animals, applications to polyploid organisms are still scarce, partly due to limited genome resources and the complexity of this system. Deep learning (DL) techniques comprise a heterogeneous collection of machine learning algorithms that have excelled at many prediction tasks. A potential advantage of DL for GP over standard linear model methods is that DL can potentially take into account all genetic interactions, including dominance and epistasis, which are expected to be of special relevance in most polyploids. In this study, we evaluated the predictive accuracy of linear and DL techniques in two important small fruits or berries: strawberry and blueberry. The two datasets contained a total of 1,358 allopolyploid strawberry (2n=8x=112) and 1,802 autopolyploid blueberry (2n=4x=48) individuals, genotyped for 9,908 and 73,045 single nucleotide polymorphism (SNP) markers, respectively, and phenotyped for five agronomic traits each. DL depends on numerous parameters that influence performance and optimizing hyperparameter values can be a critical step. Here we show that interactions between hyperparameter combinations should be expected and that the number of convolutional filters and regularization in the first layers can have an important effect on model performance. In terms of genomic prediction, we did not find an advantage of DL over linear model methods, except when the epistasis component was important. Linear Bayesian models were better than convolutional neural networks for the full additive architecture, whereas the opposite was observed under strong epistasis. However, by using a parameterization capable of taking into account these non-linear effects, Bayesian linear models can match or exceed the predictive accuracy of DL. A semiautomatic implementation of the DL pipeline is available at https://github.com/lauzingaretti/deepGP/.

## Introduction

Deep learning (DL) techniques comprise a heterogeneous collection of machine learning algorithms which have excelled at many prediction tasks, and this is a very active area of research (Min et al., 2017; Pattanayak, 2017; Namin et al., 2018). All DL algorithms employ multiple neuron layers and numerous architectures have been proposed: multiple layer perceptrons (MLPs), recurrent neural networks (RNNs), convolutional neural networks (CNNs) (LeCun et al., 2015) and others. DL is relatively straightforward to implement (https://keras.io/why-use-keras/), but optimum performance depends on an adequate hyperparameter choice, which is not trivial and requires considerable computational resources (Young et al., 2015; Chan et al., 2018). Although previous, limited evidence does not show a consistent advantage of DL over penalized linear methods for genomic prediction (GP) purposes (González-Recio et al., 2014; Ma et al., 2017; Bellot et al., 2018; Montesinos-López et al., 2018a; Montesinos-López et al., 2018b; Montesinos-López et al., 2019a), more efforts are needed to fully understand the behavior and potential constraints and capabilities of DL in GP scenarios.

Genomic selection (GS) is the breeding strategy consisting in predicting complex traits using genomic-wide genetic markers. The idea was developed to overcome the limitations of marker-assisted selection (MAS) and was formalized by Meuwissen et al. (2001). While MAS establishes a model with only the markers with significant associations, genomic selection includes all, or most available markers, for GP, irrespective of their effect and its significance. Due to the decrease in genotyping costs, genomic selection is becoming the standard tool in many plant and animal breeding programs (Bernardo, 2008; González-Camacho et al., 2012; Crossa et al., 2013b; Meuwissen et al., 2013; Wiggans et al., 2017). There is an increasing number of successful applications of genomic selection in diploid and polyploid organisms where its use has generated important genetic gains by improving the accuracy of breeding value prediction and dramatically reducing generation intervals (Crossa et al., 2013a; Castillo-Juárez et al., 2015; Duangjit et al., 2016; Juliana et al., 2019; de Bem Oliveira et al., 2019).

In any scenario, GP poses statistical challenges since the number of markers is usually much larger than the number of individuals, i.e., the so-called large *p* (number of features) small *n* (sample size) paradigm (de los Campos et al., 2013; Pérez and De Los Campos, 2014). In this context, statistical methods require either shrinkage, variable selection, or a combination of both (Tibshirani, 1996). Most GP methods are based on linear models, such as Genomic Best Linear Unbiased Prediction (GBLUP) (VanRaden, 2008), the Bayesian GP family (Meuwissen et al., 2001; Pérez and De Los Campos, 2014), or LASSO (Tibshirani, 1996). In GBLUP, all marker effects are assumed to be normally distributed with equal variance and a homogeneous shrinkage is induced, whereas Bayesian models are more flexible and differential shrinkages and/or variable selection can be applied to distinct marker subsets. Note that these methods are linear and, in contrast to DL, have not been designed to model non-additive genetic effects (such as dominance or epistasis); however, these effects can be incorporated in the model with appropriate parameterizations.

One potential advantage of DL for GP over standard methods is that the whole genetic merit, including all non-additive effects, can potentially be predicted without the need to partition all effects. This is an interesting property for clonally propagated outcrossing species, because genomes can be asexually reproduced from single plants once the desirable individual is found. It should also be a promising strategy in polyploids, although their complex genetic structure has delayed the availability of whole genome markers and of specific analytic tools for, e.g. SNP calling (Slater et al., 2016; Gezan et al., 2017; Bourke et al., 2018). A few studies have demonstrated the potential advantages of GS in allo and autopolyploids (Gezan et al., 2017; Enciso-Rodriguez et al., 2018; Nyine et al., 2018; Amadeu et al., 2019; de Bem Oliveira et al., 2019; Juliana et al., 2019; Zingaretti et al., 2019), although its implementation is still in its infancy.

When non-additive effects are investigated, there are two important points that need to be considered for higher ploidy levels: i) there is a portion of the intra-locus allele interaction (i.e., dominance) that is passed to the progeny (particularly full-sibs), and ii) the definition of non-additive effects is more complex than in diploids as higher order interaction exist (Osborn et al., 2003). Thus, methodologies that could model the whole genetic merit without restrictive assumptions could facilitate and improve the prediction for polyploid species, making DL an attractive choice for genomic prediction. In practice, DL aims at predicting the whole genetic merit, including interactions irrespective of their origin.

Among the polyploid species, strawberries (*Fragaria x ananassa*) and blueberries (*Vaccinium corymbosum*) are considered two of the most important soft fruit commodities. Considered a rich source of vitamins and minerals, fruit markets for both species have experienced a global increase in production and consumption over the past decade (https://www.nass.usda.gov/Publications/Todays_Reports/reports/ncit0619.pdf). To ensure that production and fruit quality meet the global demand, genetic improvement, and particularly GP, has a role to play in maximizing the utility, diversity, and yield of resources. In this sense, previous experimental assessments performed in blueberry (Amadeu et al., 2019; de Bem Oliveira et al., 2019) and strawberry (Gezan et al., 2017) have proven the feasibility of incorporating genomic selection to either accelerate the pace or improve the efficiency of breeding programs. From a genetic standpoint, one important difference between both species is its inheritance pattern. Cultivated strawberry (*Fragaria x ananassa)* is an allo-octoploid hybrid plant originated by cross between two wild octoploid species *F. chiloensis* and *F.virginiana* (Hancock et al., 2008) both descendants of *Fragaria* diploid species; referred as allopolyploids, meiosis is mainly dictated by preferential pairing, exhibiting a diploid-like (or disomic) segregation. In contrast, blueberry is a tetraploid organism originated from genome duplication within the same species. In autopolyploids, the meiotic pairing is mainly described by forming either random bivalents or multivalent during the division. Since the molecular mechanisms in auto and allopolyploids are quite complex, comparing new algorithms is a relevant issue to the prospect of GP in these and other polyploid species.

In this study, we evaluated the performance of deep learning for genomic prediction in two important horticultural species: allo-octoploid strawberry and auto-tetraploid blueberry. We complement the empirical study with simulations to understand better the impact of genetic architecture on DL performance. Given the complexity of implementing DL, we also provide a guideline on best practices for hyperparameter tuning and evaluate its importance in terms of predictive ability. To facilitate reproducibility of these methods, a python-based package for semiautomatic DL implementation, including auto and allopolyploid organisms have been made available at https://github.com/lauzingaretti/deepGS/.

## Materials and Methods

### Plant Material and Genotypes

Predictive performances were compared in two polyploid species (blueberry and strawberry), for a series of traits with presumably contrasting genetic architecture. A summary of both experimental data sets is presented in the **Table 1**.

**Table 1:** Summary of blueberry and strawberry experimental data sets used in this paper.

	Strawberry (allopolyploid)	Blueberry (autopolyploid)
**Ploidy**	2n = 8x = 112	2n = 4x = 48
**No. observations**	1,358 (1,233 unique genotypes)	1,802
**No. SNPs**	9,908	73,045
**Traits analyzed**	Soluble solid content (brix) Average fruit weight (AveWtT) Total marketable weight (MktWtT) Early marketable yield (MktWtE) Percentage of culled fruit (CullsTPer).	Firmness Fruit Size Weight Yield Scar
**Main reference**	Gezan et al. (2017)	Amadeu et al. (2019) and de Bem Oliveira et al. (2019)

Regarding strawberry, we used 1,233 unique genotypes which correspond to five advanced selection trials (T2, T4, T6, T8, and T10) from the strawberry breeding program at the University of Florida, Institute of Food and Agricultural Sciences (USA). These advanced trials were planted in five consecutive seasons and were given an even code starting with season 2013–2014 as T2 and ending with season 2018–2019 as T10. The number of lines in each trial was 217, 240, 236, 272, and 393 for T2, T4, T6, T8, and T10, respectively. Some of the genotypes in the last trial T10 were already tested in earlier trials, making the total number of observations sum up to 1,358 (instead of 1,233). Plants were genotyped with the Axiom IStraw90 SNP array (Bassil et al., 2015). After quality control, in which those markers with minor allele frequencies (MAF) < 5% and with missing marker data > 5% were eliminated, 9,908 polymorphic SNP markers were available. A total of five yield and fruit quality traits were evaluated in each trial: soluble solid content (brix), average fruit weight (AveWtT), total marketable yield (MktWtT), early marketable yield (MktWtE), and percentage of culled (unmarketable) fruit (CullsTPer). Additional details for T2 and T4 can be found in Gezan et al. (2017).

The blueberry population used in this study encompasses one cycle of the University of Florida blueberry breeding program's recurrent selection and comprised 1,802 lines from 117 full-sib families. The population was originated from 146 parents that presented superior phenotypic performance (cultivars and advanced stage of breeding). Individuals were evaluated for five yield and fruit quality-related traits: firmness, fruit size, weight, yield, and picking scar, which were collected during two production seasons. Phenotypes were pre-corrected for fixed year effects, as detailed in Amadeu et al. (2019) and de Bem Oliveira et al. (2019). A total of 73,045 SNPs was obtained using sequence capture by Rapid Genomics (Gainesville, FL), after aligning the reads against the high-quality “Draper” genome assembly (Colle et al., 2019) as described in Benevenuto et al. (2019). Marker filtering followed these criteria: biallelic, mean coverage > 40, minimum allele frequency > 0.01; maximum missing data = 0.5%; minimum quality = 20. Also, individuals with more than 50% missing data were removed, missing genotypes were simply imputed with the mean. Tetraploid genotypes were called and the allele dosages were inferred with the updog R package (Gerard et al., 2018). Standard genotype calling with updog allows inferring genotypes according to the number of allele copies, and genotypes can be coded say 0,1,2,3,4. In addition, as in de Bem Oliveira et al. (2019), here we considered a set of “diploidized” genotypes that were obtained pooling all heterozygous genotypes in a single class, i.e., genotypes above 0,1,2,3,4 can be recoded as 0,1,1,1,2. The rationale is that there can be incertitude on the exact number of allele copies in heterozygous genotypes.

The GP methods evaluated in this study were assessed by true validation, which was obtained by splitting data into a training and a validation dataset. In the strawberry dataset, we considered that predicting performance of the last stage lines (T10) is the most interest application for the industry and therefore the population was divided between training (T2, T4, T6, and T8 trials) and validation (T10) subsets with 965 and 393 lines, respectively. In the case of blueberry data, all samples were equally important and 30% of randomly sampled genotypes were assigned to the validation set. Predictive ability (PA) was defined as the correlation between observed and predicted phenotypes in the validation set; prediction was computed from parameters estimated in the training dataset only.

### Genetic Structure and Heritability Inference

Potential genetic structure was assessed by principal component analysis (PCA) using all genotypes. Since genetic architecture may have an impact on GP performance and on the optimum GP model (Daetwyler et al., 2013), additive and non-additive genetic features were assessed by computing variance components from the model:

(1) y=μ1+a+d+e+ε

where the vector ***y*** represents the adjusted phenotype, ***μ***1 is the intercept, $a∼N\left(0,A\sigma_{a}^{2}\right),\;\;d∼N\left(0,D\sigma_{d}^{2}\right)$ and $e∼N\left(0,E\sigma_{e}^{2}\right)$ are the additive, dominant and epistatic effects, respectively, and $\varepsilon ∼N\left(0,I\sigma_{\varepsilon }^{2}\right)$ is the residual component. Matrices **A** and **D** were obtained using AGHmatrix package (Amadeu et al., 2016) for both strawberry (as diploid) and blueberry (autotetraploid) species. For diploids, **A** and **D** were computed using VanRaden (2008) and Vitezica et al. (2013) methods, respectively. In fact, $A=\frac{ZZ^{\prime }}{2\sum_{j}p_{j}\left(1-p_{j}\right)}$ where **Z** is the matrix that contains the centered individual genotype values and $D=\frac{MM^{\prime }}{4\sum_{j}{\left[p_{j}\left(1-p_{j}\right)\right]}^{2}}$ is the dominance matrix, where the **M** elements are $-2p_{j}^{2},\;2p_{j}\left(1-p_{j}\right),\;-2{\left(1-p_{j}\right)}^{2}$ for genotypes 0, 1, and 2, respectively. In the case of ploidy = 4, **D** was obtained as in Slater et al. (2016). The epistatic matrix (**E**) considered is the Hadamard product of additive effects, i.e. A⊙A  (Henderson, 1984) Posterior distributions of genetic parameters were obtained using Reproducing Kernel Hilbert Spaces (RKHS) regression with BGLR package (Pérez and De Los Campos, 2014). The additive, dominance and epistatic ratios were estimated as: ${\hat{h}}_{a}^{2}=s_{a}^{2}/\left(s_{a}^{2}+s_{d}^{2}+s_{e}^{2}+s_{\varepsilon }^{2}\right),{\hat{\;h}}_{d}^{2}=s_{d}^{2}/\left(s_{a}^{2}+s_{d}^{2}+s_{e}^{2}+s_{\varepsilon }^{2}\right)$ and ${\hat{h}}_{e}^{2}=s_{e}^{2}/\left(s_{a}^{2}+s_{d}^{2}+s_{e}^{2}+s_{\varepsilon }^{2}\right)$; where$s_{i}^{2}$ the *i*^th^ mean posterior estimates of *σ*^2^ as in Equation 1. We used both training and validation datasets combined in this stage, since this is purely a descriptive analysis and the values obtained are not employed in the later prediction stages.

### Penalized Linear Methods

We compared the prediction performance of DL models with two well-established linear methods: Bayesian Lasso (BL, Meuwissen et al., 2001) and Bayesian Ridge Regression (BRR, Gianola, 2013). In these models, the trait can be expressed as:

(2) y=μ1+g+ε

where ***μ***1 is the overall mean, ***g***=***Xβ, X*** is the genotypes' matrix and ***β*** is a vector of marker effects. In BRR, prior distributions of marker effects ***β*** are $N\left(0,I\sigma_{\beta }^{2}\right)$, whereas the prior distributions for ***β*** in BL have a Laplace distribution, i.e., $p(\beta |\;\lambda ,\sigma_{\varepsilon }^{2})=\frac{\lambda }{2\sigma_{\varepsilon }^{2}}\text{exp}\left(-\left|\beta \right|\frac{\lambda }{\sigma_{\varepsilon }^{2}}\right)$. Note that the Laplace distribution does not remove markers so, contrary to its frequentist counterpart, BL is not a variable selection approach. Each model was fitted by using only phenotypes from the training subset. The models were run using the BGLR package (Pérez and De Los Campos, 2014) with a Gibbs sampler algorithm for a total of 6,000 cycles, discarding the first 1,000 samples for burn-in.

The above parameterization assumes additivity of effects, although linear models can address non-linear relationships if properly parameterized. Non-linear interactions can be modeled by expressing ***g*** (Equation 2) in a general way, i.e., ***g* **=** *Ω ω*** where **Ω** (centered and scaled) is a matrix of dummy variables that indicates the number of copies of the reference allele ranging from 0 to the ploidy level (Slater et al., 2016; Enciso-Rodriguez et al., 2018). This model is, in principle, a good parameterization to account for non-linear interactions and we will refer to it as BRR general model (BRR-GM), since Bayes ridge regression was used. For more details, see Enciso-Rodriguez et al. (2018) and Amadeu et al. (2019).

Non-linearity can also be managed by means of RKHS regression (Gianola et al., 2006) as an alternative to a linear regression for capturing complex interactions. This model considers ***g*** in Equation 2 as $N\left(0,K\sigma_{g}^{2}\right)$ with $K\left(x_{i},x_{i^{\prime }}\right)=\text{exp}\left(-\frac{h{\left||x_{i}-x_{i^{\prime }}|\right|}^{2}}{p}\right),$ a kernel function where *h* is de bandwidth parameter controlling how fast the covariance function drops with the distance between pairs of markers and $||x_{i}-x_{i}|{|}^{2}$ is the Euclidean distance between any two pairs of genotypes. This parameterization induces a general matrix of genetic covariance between markers. The key point here is that the kernel can model non-linear relationships because it is a non-linear transformation of the distances between the input variables. Empirical evidence confirms that it is an accurate approach to predict phenotypes of complex traits (Gianola et al., 2008; de los Campos et al., 2009; de Los Campos et al., 2010). BRR-GM and RKHS were only implemented for strawberry and simulated scenarios, since it was in strawberry where we found the trait with the largest epistasis component, as described below.

### Deep Learning (Convolutional Neural Networks)

**Figure 1:**
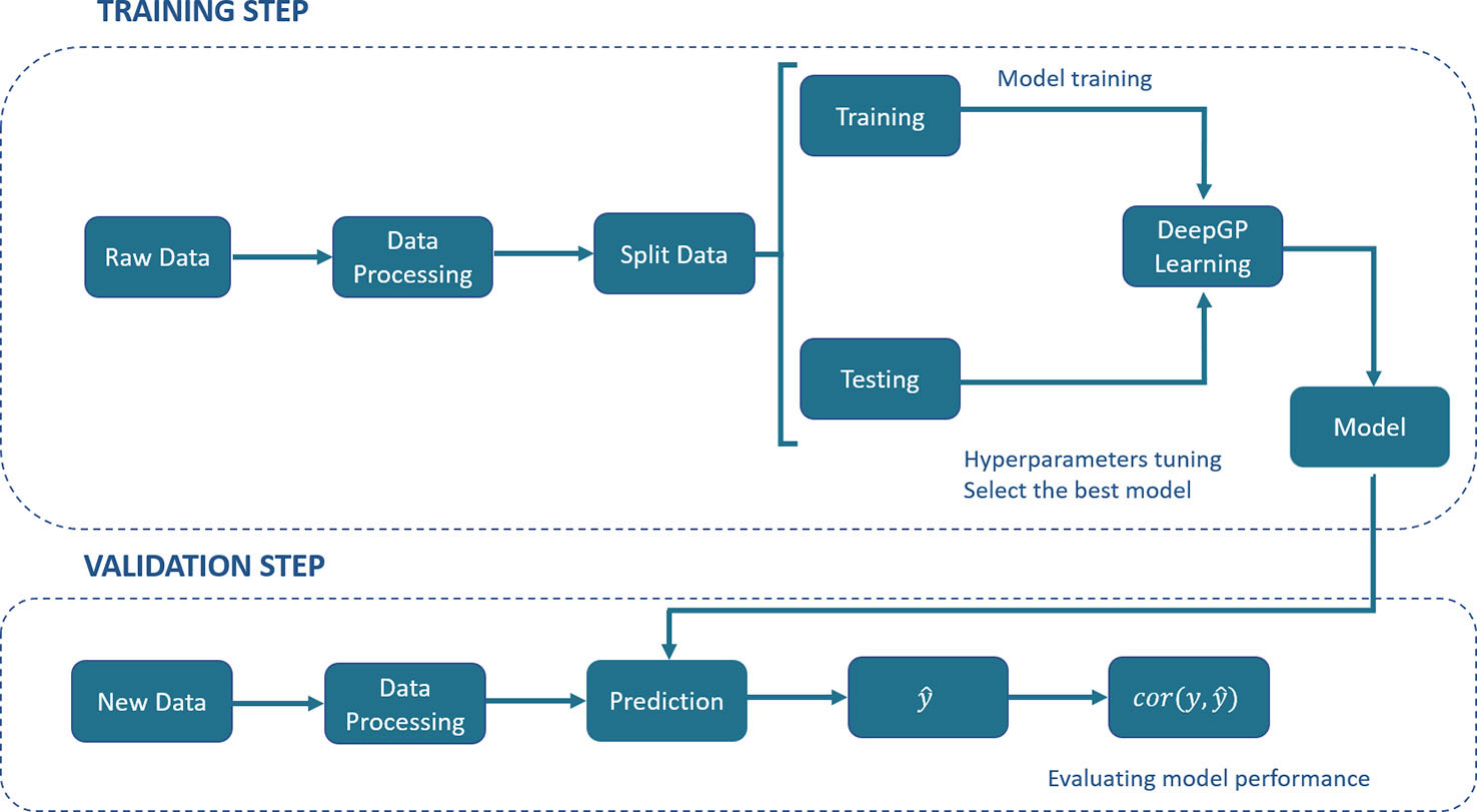
A generic deep learning (DL) pipeline for genomic prediction (GP) purposes. The general process includes the training and validation steps. In the training step, data are split into training and testing, DL hyperparameters are optimized by internal cross-validation with the test set and the model with the best predictive ability (PA) is chosen. In the validation step, the model PA is evaluated using a new set of data.

DL has been described as a universal learning approach able to solve supervised, semi-supervised and unsupervised problems. Several DL architectures have been proposed, such as MLPs, RNNs, CNNs, Generative Adversarial Networks (GANs) and Reinforcement Learning (RL). **Figure 1** shows a generic pipeline to evaluate DL in a GP context.

**Figure 2:**
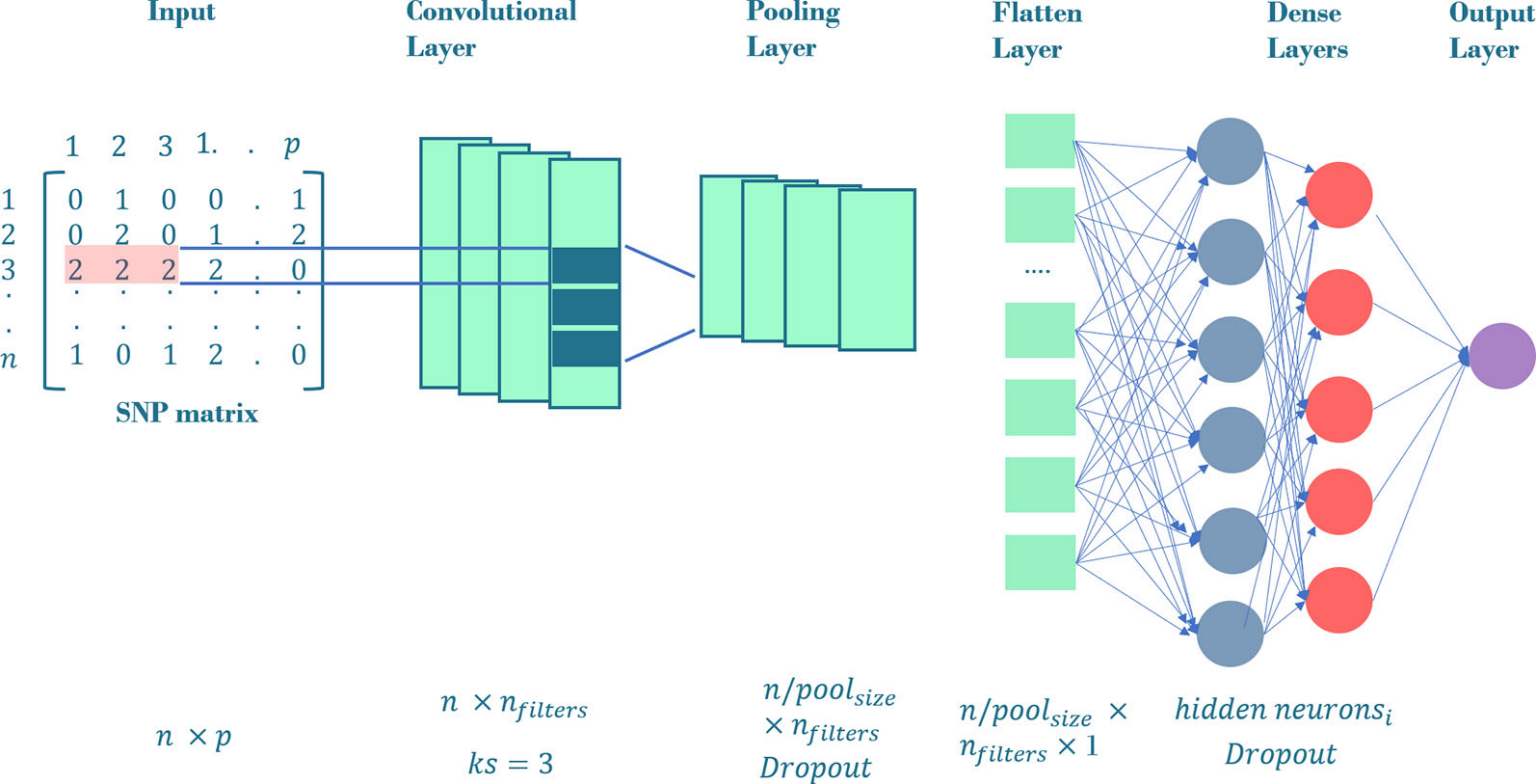
General CNN architecture employed in our workflow. The input layer is a SNP matrix of size *n* x *p*, where *n* is the number of training set and *p*, the number of SNPs. The convolutional layer consists on a *n_filters_* convolution followed by a max-pooling layer with pool_size_ = 3 and an optional dropout. The outputs of max-pooling layer are joined together into one vector by flattening. All the neurons in the flatten layer are fully connected to the first dense layer. We tune the network using *i* dense layers with a variable number of hidden neurons in the respective hidden layers. The output of these dense layers is the prediction layer that uses linear function as activation. The neurons in convolutional and dense layers use relu, tanh or linear function as activations.

In our previous experiment (Bellot et al., 2018), CNNs were the best performing methods and therefore are the only ones discussed here. The advantage of CNNs in a GP context is that they can model the correlation between adjacent input variables, that is, linkage disequilibrium between nearby SNPs. This is done via a mathematical operation called convolution (Goodfellow et al., 2016). A typical CNN is made up of “convolutional layers”, “pooling”, “flatten” and “dense” fully connected layers (**Figure 2**). In the “convolutional layer”, an operation called convolution is performed along the input of predefined width and strides, which are known as “kernel” and “filter” in the DL jargon, respectively. From a mathematical view, a convolution *s(t)* is a function that can be defined as an “integral transform” (Widder, 1954):

(3) s(t)=(f*k)(t)=ʃk(t−x)f(x)dx

where one of the functions (*k* or *f*) in Equation (3) must be a kernel. Assuming that the kernel is represented by *k*, the convolution is the transformation of *f* (input data in the DL context) into *s(t)*. The operation is just the weighted sum of an infinite number of copies *f* shifting over the kernel. The discrete version of Equation 3 follows naturally as:

(4) $$s\left(t\right)=\left(f*k\right)\left(t\right)=\sum_{x}k\left(t-x\right)f\left(x\right)$$

One of the main advantages of convolution networks is their capability to reduce the number of operations, i.e., the hyperparameters to be estimated. As usual, an activation function (generally non-linear) is applied after each convolution to produce the output layer. Finally, “pooling” layers reduces dimension and achieves a smoother representation, summarizing adjacent neurons by computing their maximum or mean.

### Hyperparameter Optimization

Since DL depends on numerous parameters that influence performance, optimizing hyperparameter can be a critical unresolved step, which relies heavily on heuristics. Hence, it is surprising that many DL applications in GP have not paid enough attention to this problem (Ma et al., 2017; Montesinos-López et al., 2018b; Montesinos-López et al., 2019b). Several approaches have been proposed for hyperparameter tuning (e.g., Bellot et al., 2018; Cho and Hegde, 2019; Le et al., 2019; Rajaraman et al., 2019; Yoo, 2019). Here, DL architectures were optimized using Talos (Autonomia Talos, 2019), which works combining all parameters in a grid. Talos can choose the best model either maximizing the predictive accuracy or minimizing the error; the former criterion was employed here. Since the approach can be expensive as the number of hyperparameters increases, a random search is the best strategy in practice. This rule evolves a list of CNN models for each phenotypic trait. We optimized the following hyperparameters (values considered within parentheses): activation function (relu, tanh, linear), number of filters (16, 32, 64, 128), regularization (i.e., weight decay in DL terminology, 0, 0.1, 0.01, 0.001), learning rate (0.1, 0.01, 0.001, 0.0025), number of neurons in fully connected layer (4, 8, 12, 16), number of hidden layers (1,5,10), and dropout (0, 0.01, 0.1, 0.2).

Talos output is the accuracy for each hyperparameter combination; we then used hyperparameter values as independent variables and accuracy as target variable to run a random forest algorithm, which allowed us to compute the hyperparameter value importance, measured as the decrease in Gini's coefficient when adding the given hyperparameter. This hyperparameter importance can be then used as guide to improve interpretability. The R package randomForest (Liaw and Wiener, 2002) was employed for this analysis.

The DL algorithms used in this study were implemented in Keras (Chollet, 2015) and Tensorflow (Abadi et al., 2015) and run on a GPU equipped Linux workstation. A generic script is publicly available at https://github.com/lauzingaretti/deepGS/.

### Simulation

We studied the impact of genetic architecture on prediction performance by simulation using the actual observed strawberry genotypes, assessing predictive performance with the same T10 strawberry genotypes (and genotypic data) as in the real experiment, except that phenotypic responses were simulated. Three contrasting genetic architectures were considered:

1. Additive: 200 randomly chosen SNPs were considered as causal loci. No dominance was simulated. Total individual genetic value was the sum of effects across loci.
2. Epistatic: 100 epistatic pairs of SNPs were randomly sampled. Epistasis was multiplicative by pairs, i.e., the genotype was the product of individual genotypes in each pair. Total genetic value was the sum of effects across pairs of loci.
3. Mixed: 80 individual additive SNPs and 60 epistatic SNP pairs were randomly chosen. Total genetic value was the sum of effects across pairs of loci and individual additive loci.

Allele substitution effects were sampled from a gamma distribution Γ(α = 1, β = 0.2). The trait was obtained adding the genetic value to an environmental normal residual. Environmental variance was chosen such that broad-sense heritability was set to 0.50. For each genetic architecture, five replicates were run. We compared BRR, BRR-GM, RKHS, and DL. DL architectures were specifically optimized to each phenotypic trait, since no universal architecture is able to make accurate predictions for all cases.

## Results

### Population Structure and Genetic Parameters

No clear population structure was observed, neither in the strawberry nor in the blueberry datasets (**Figure S1**). Note that genetic relationships between trials in strawberry data are rather uniform, irrespective of whether they are successive seasons or not. This, together with the fact that little genotype by environment (or year) interaction was observed (Gezan et al., 2017), suggests a favorable scenario for GP.

**Figure 3:**
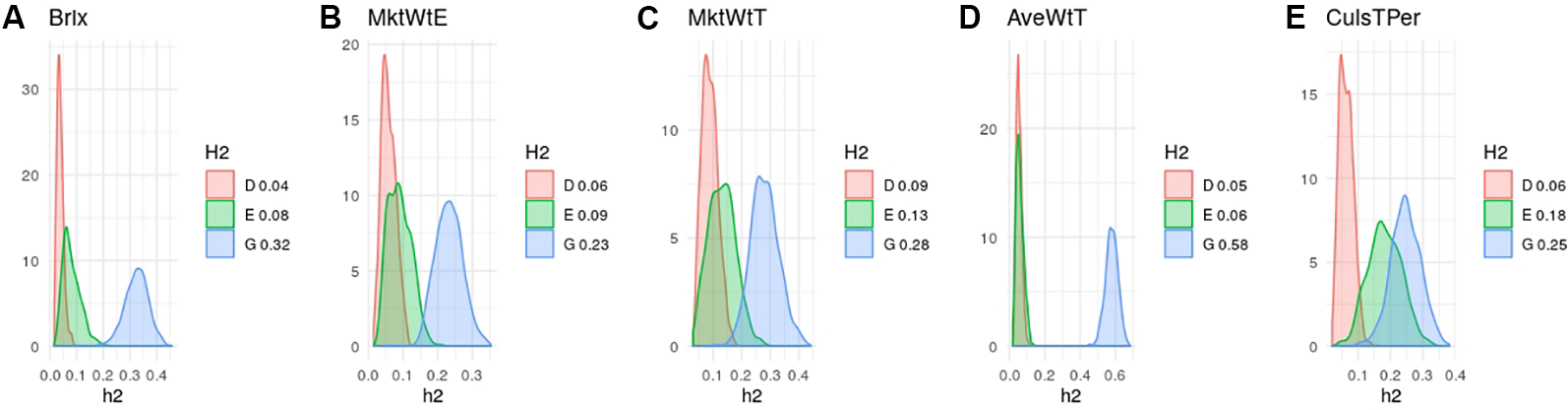
Posterior distributions of additive (blue), dominant (red), epistasis (green) fractions of variance in octoploid strawberry: **(A)** soluble solid content (brix); **(B)** early marketable yield (MktWtE); **(C)** total marketable yield (MktWtT); **(D)** average fruit weight (AveWtT); and **(E)** percentage of culled fruit (CullsTPer). Note the scale may vary along traits.

**Figure 4:**
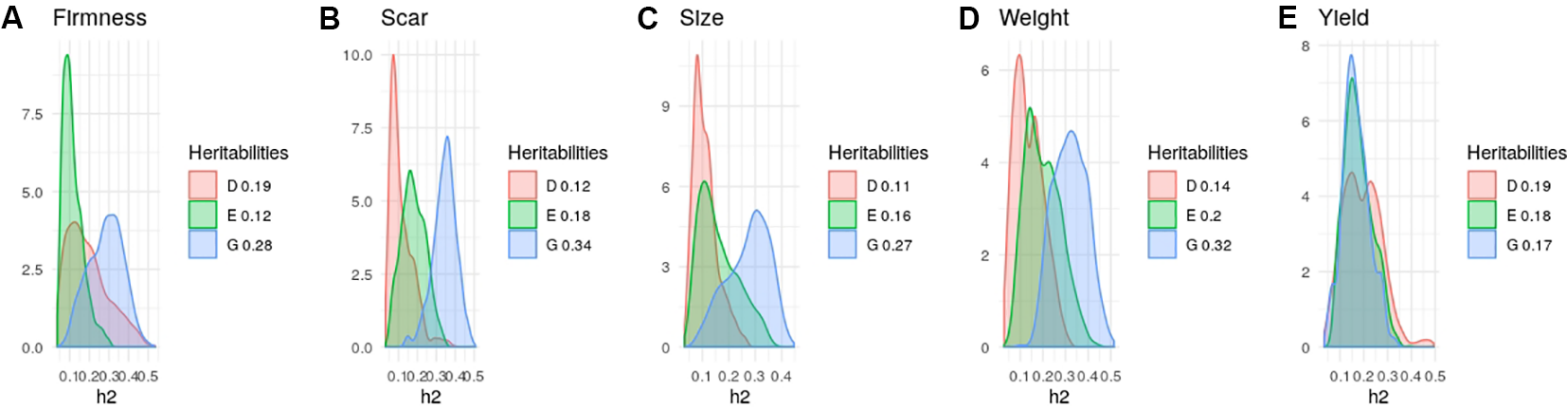
Posterior distributions of additive (blue), dominant (red), epistasis (green) fractions of variance in blueberry obtained with the tetraploid genotypes: **(A)** Firmness; **(B)** Scar; **(C)** Size; **(D)** Fruit Weight, and **(E)** Yield. Note the scale may vary along traits.

Heritability estimates in strawberry are slightly different from those obtained in the same material by Gezan et al. (2017) since here we used additional data and we removed genotypes tested since here we used additional material and we removed genotypes tested more than once on different seasons. Nevertheless, in agreement with previous results (Amadeu et al., 2019; de Bem Oliveira et al., 2019; Gezan et al., 2017) narrow-sense heritabilities were moderate, ranging from 0.25 to 0.35 for most strawberry (**Figure 3**) and blueberry (**Figure 4**) traits, except for strawberry average fruit weight $\left(h_{a}^{2}=\;0.58\right)$ The degree of dominance found was quite low in general, especially in strawberry. An exception was blueberry yield, where dominant and epistatic variances were similar to the additive variance (**Figure 4E**). A remarkable case is percentage of culled fruit (CulsTPer) in strawberry, where the epistatic ratio (18%) was only slightly smaller than the additive one (25%, **Figure 3E**).

### Hyperparameter Importance

**Figure 5:**
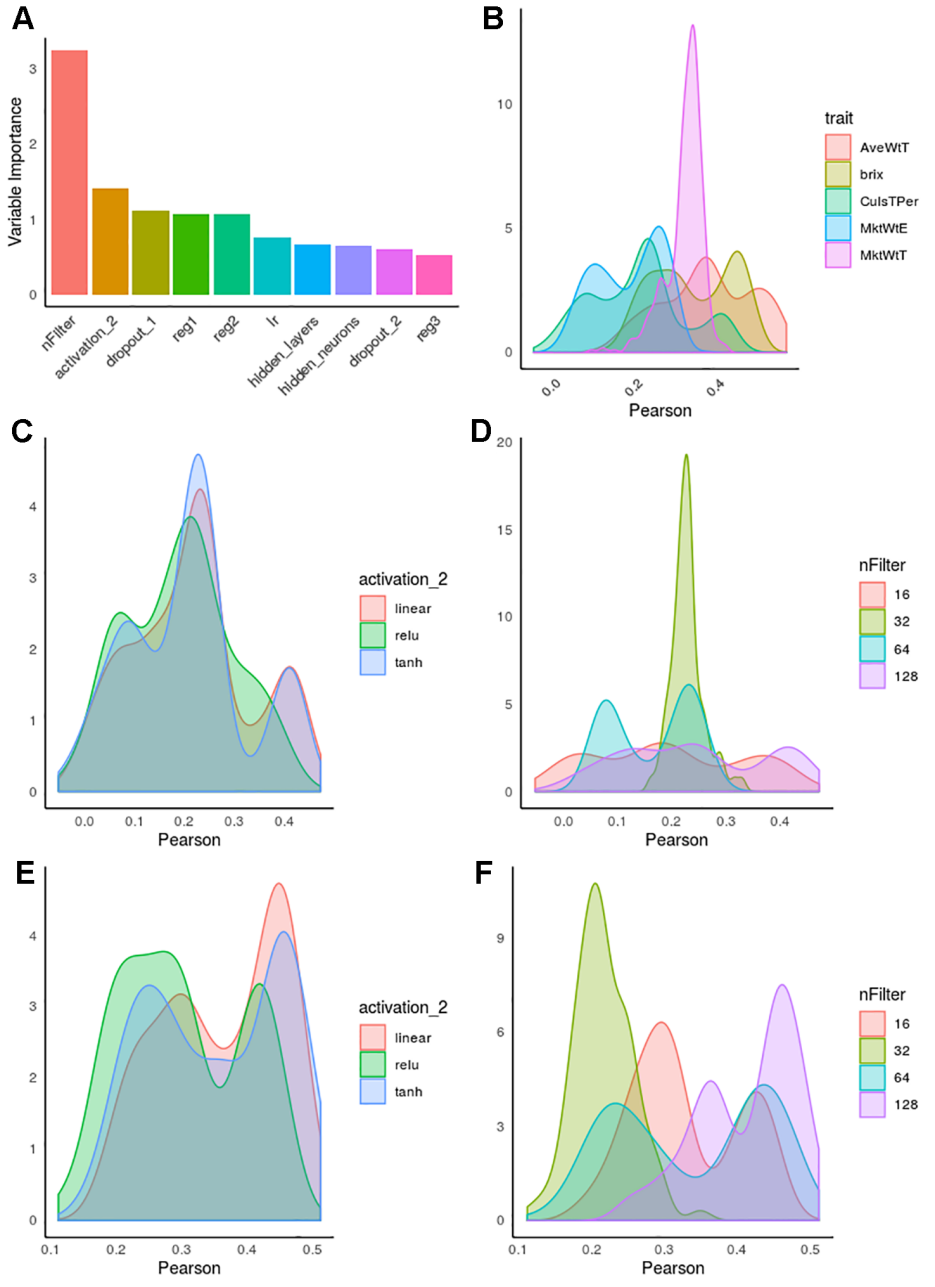
Hyperparameter influence on predictive accuracy in strawberry. Accuracy is defined as correlation between observed and predicted phenotypes by internal cross-validation. **(A)** Hyperparameter importance obtained from a random forest algorithm. nFilter: number of filters in the convolutional layer, activation_2, activation function in layer 2; reg_i, regularization in i-th layer; dropout_i, dropout rate in i-th layer; lr, learning rate; hidden_neurons, number of neurons in inter-mediate layers; hidden_layers, number of intermediate layers. **(B)** Distribution of accuracies along hyperparameter combinations for each phenotype. **(C)** Accuracies as a function of activation function for percentage of culls. **(D)** Accuracies as a function of number of filters for percentage of culls. **(E)** Distribution of accuracies as a function of activation for brix. **(F)** Distribution of accuracies as a function of number of filters for average fruit weight (AveWtT).

CNN hyperparameters were optimized for each strawberry trait separately. **Figure 5A** shows the importance of each hyperparameter obtained from random forest by regressing the model predictive accuracies (obtained by an inner cross-validation) on all hyperparameter values combinations. Interestingly, the number of filters was overall the most relevant factor, whereas other factors such as learning rate, whose importance has been claimed in the literature as critically important (Maas et al., 2013; Bawa and Kumar, 2019; Feng and Lu, 2019), played only a minor role. We also observed that the effect of each hyperparameter depends on the layer, e.g. regularization or dropout were more important in first than in deep layers.

In **Figure 5A**, the “trait” effect was excluded since it cannot be controlled by the experimenter, although it was by far the most influential variable. This is illustrated in **Figure 5B**, which shows the distribution of accuracies for each trait studied. Not only maximum accuracies varied across trait, the profiles were also extremely different, usually multimodal. This suggests interactions between hyperparameter combinations, and it also indicates that trait–specific optimization should be performed whenever possible.

**Figure 5** illustrates the kind of complex interactions that we observed in hyperparameter optimization. For instance, **Figures 5C, E**, show the distinct influence of activation functions in percentage of culled fruit (**Figure 5C**) and brix (**Figure 5E**). Although “relu” activation function has been suggested as the activation of choice in recent DL literature (Maas et al., 2013; Pouladi et al., 2016), here we observed that linear or even sigmoid-like hyperbolic tangent (tanh) seemed to be a safer choice overall. It is relevant to note that interactions were clearly observed for some hyperparameters, such as the number of filters. For CulsTPer, either 16 or 128 filters resulted in optimum accuracies, although they were also associated with the worst hyperparameter combinations. In contrast, either 32 or 64 filters are to be preferred for average weight in strawberry (**Figure 5F**).

The final sets of hyperparameters for strawberry and blueberry phenotypes are indicated in **Tables S1** and **S2**, respectively. Overall, our study shows that shallow architectures are more competitive than deep architectures in terms of PA, since the majority of models only included one CNN layer. The number of filters -in combination with dropout- has a large effect in the PA, but is highly dependent of the trait. For instance, all optimal architectures for strawberry contain 128 convolutions, but this is much more variable in the case of blueberry, with a range between 16 and 128 convolutional operations. As for the fully connected layers, the situation is less clear, and no obvious pattern is observed. We can highlight some characteristics though, for example, the number of hidden fully connected layers is quite variable, but only a few neurons (4, 8, 12) are preferable in most of the architectures. As also reported by Waldmann (2018), combining weight decay and dropout regularization is an efficient option to increase PA. Finally, the best overlapping stride was 1 and optimum window size was 3 in the convolutional layer, confirming Bellot et al. (2018) results.

### Comparing Deep Learning With Bayesian Penalized Linear Models

**Figure 6:**
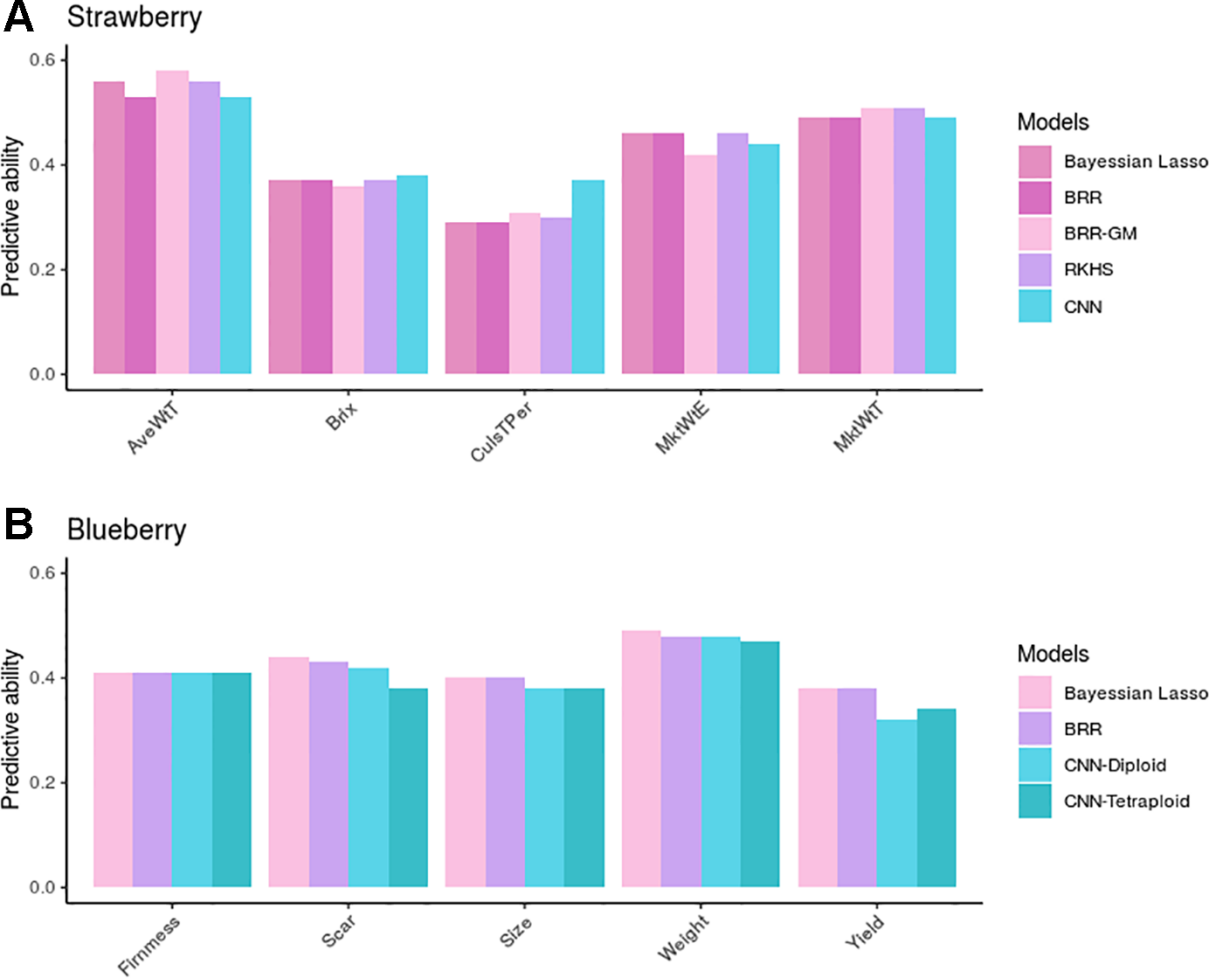
Predictive ability (PA) measured as correlation between observed and predicted phenotypes in the validation dataset in strawberry **(A)** and blueberry **(B)**. Bayesian linear models (lasso and BRR) PAs in blueberry were computed with tetraploid genotypes, but were almost identical to those obtained with the diploidized ones.

**Figure 6A** shows observed predictive abilities for each of the five GP methods compared: BL, BRR, BRR-GM, RKHS, and CNNs in strawberry. When averaged over traits in the strawberry species, PAs were 0.43, 0.43, 0.44, 0.44, and 0.44 for each of the five methods, respectively. By trait, the BRR-GM was best in AveWtT prediction, BL, BRR, and RKHS for MktWtE, RKHS, and BRR-GM for MkWtT, whereas CNN performed best in brix and percentage of culled fruit. In all, nevertheless, there were no important differences between methods except in percentage of culled fruit. For this trait, CNN was ~20% better than any linear model method. Interestingly, this trait was also the one with the largest epistatic component and exhibited a modest additive component (**Figure 3E**).

As for the blueberry phenotypic traits, we found no differences between GP methods BL and BRR (average PA = 0.42), whereas CNNs were somewhat underperforming (average PA = 0.40). The most remarkable result in blueberry is that CNN performance was barely affected by the ploidy level employed to build the genetic relationship matrix. In fact, the “diploid” option seemed more robust than the tetraploid one, except in fruit yield, the only trait that was measured using a rating scale.

### Simulation Study

**Table 2** presents the main simulation results and **Table S3**, the CNN architectures used for computing the PA in each replicate. Some interesting remarks can be made from these simulations. First, although biased, the variance component estimates do detect whether epistasis is important: $h_{e}^{2}$ estimates are larger than the narrow-sense heritability in the presence of complete epistasis. Results are far less clear when only a fraction of loci show epistasis. But the most relevant result is that, as we hypothesized, predictive accuracies of CNN and additive penalized methods were affected by genetic architecture. BRR and RKHS were better than CNNs for the pure additive architecture, whereas the opposite was observed with pure epistasis. However, BRR-GM, which accounts for non-linear relationships, was better than either CNNs or pure additive linear models in most of the studied cases.

**Table 2:** Posterior distribution means of variance component estimates $\left({\hat{h}}_{2}\right)$ and predictive ability (in simulated data using Bayes Ridge Regression (BRR), general model BRR (BRR-GM), Reproducing Kernel Hilbert Space regression (RKHS), and Convolutional Neural Networks (CNN).

Replicate	Architecture	Genetic parameter estimates			Predictive ability (PA)			
		${\hat{h}}_{a}^{2}$	${\hat{h}}_{d}^{2}$	${\hat{h}}_{e}^{2}$	**BRR**	**BRR-GM**	**RKHS**	**CNN**
**1**	Additive	0.29	0.16	0.06	0.57	0.60	0.57	0.59
**2**	Additive	0.16	0.21	0.06	0.35	0.43	0.35	0.32
**3**	Additive	0.26	0.25	0.05	0.52	0.58	0.51	0.51
**4**	Additive	0.24	0.23	0.06	0.42	0.52	0.43	0.40
**5**	Additive	0.35	0.11	0.05	0.42	0.47	0.43	0.38
**6**	Mixed	0.17	0.19	0.06	0.33	0.44	0.33	0.30
**7**	Mixed	0.10	0.11	0.08	0.24	0.26	0.22	0.24
**8**	Mixed	0.16	0.10	0.08	0.29	0.33	0.31	0.28
**9**	Mixed	0.13	0.16	0.06	0.26	0.30	0.26	0.25
**10**	Mixed	0.22	0.07	0.07	0.40	0.42	0.40	0.43
**11**	Epistatic	0.11	0.11	0.21	0.23	0.29	0.24	0.25
**12**	Epistatic	0.11	0.11	0.33	0.31	0.37	0.32	0.34
**13**	Epistatic	0.12	0.09	0.23	0.34	0.38	0.35	0.32
**14**	Epistatic	0.05	0.13	0.21	0.21	0.34	0.23	0.28
**15**	Epistatic	0.10	0.11	0.15	0.21	0.31	0.23	0.21

## Discussion

Supervised DL methods are examples of predictive modelling, consisting of approximating a mapping function (*f*) from input (***X***) to output (***y***) variables (Hornik et al., 1990). These problems include classification or regression tasks, to use the machine learning jargon. Numerous successful applications of DL in classification contexts have been published, e.g. pattern recognition (Drayer and Brox, 2014; Liang and Hu, 2015; Işin et al., 2016; Badrinarayanan et al., 2017) and natural language processing (NLP) (Deng and Liu, 2018). The DL implementation in regression tasks is less abundant and the benefit of using these methods remains uncertain (Bellot et al., 2018; Montesinos-López et al., 2018a; Azodi et al., 2019). Most GP problems fall into the regression task due to the complex nature of quantitative traits (MacKay, 2009). So far, GP problems have been mainly addressed using penalized linear models (De Los Campos et al., 2009; Crossa et al., 2013a). More recently, the prediction of complex traits from genetic data is receiving attention from DL users (Ma et al., 2017; Montesinos-López et al., 2018a; Montesinos-López et al., 2019b). The present work aim was to study the strengths and weaknesses of applying CNN to GP problems in polyploid species. CNN networks are attractive for addressing these problems, as they can accommodate situations where input variables are distributed along a space pattern, as with the case of SNPs.

Implementing GS in polyploids is challenging. In allopolyploids, genetic analyses have been traditionally implemented assuming diploidy, taking advantage of the fact that systems present disomic inheritance. High predictive performances have been observed in a variety of allopolyploid species (e.g. cotton, strawberry, wheat) and traits (Gezan et al., 2017; Gapare et al., 2018; Juliana et al., 2019). Recently, the importance of accounting for the contribution of subgenomes— potentially expressing epistatic effects— was considered in wheat, which shed light on the importance of accounting for this source of variation within the GP models (Santantonio et al., 2019). However, the scenario is even more complex in autopolyploid species. Even with the recent advances in genotyping and sequencing technologies, the amount of genomic information, and understanding, in most autopolyploid species is still limited when compared to allopolyploid crops. One of the challenges is resolving the allelic dosage of individual locus (Bourke et al., 2018; Gerard et al., 2018). From a quantitative genetics standpoint, we emphasize that polyploid species might present higher degrees of complete and partial intra-locus interactions than diploids (Gallais, 2003; Ferrão et al., 2018). Here, the interest of investigating DL methods in polyploids is to take advantage of its non-linearity and less restrictive assumptions for GP in comparison to the traditional linear model-based methods.

Previous studies (Ma et al., 2017; Bellot et al., 2018; Montesinos-López et al., 2018a; Montesinos-López et al., 2019b) have not shown clear advantages of DL over linear model GP, as conventional models were competitive in terms of PA, with their added benefit of being faster and with more biological interpretability. However, DL could be better suited to explore non-linear components than linear models, especially when genotypes can be transmitted integrally, as occurs with asexual propagation. Certainly, the weak performance of classical additive models in the presence of non-additive variance (e.g. **Figure 6** for percentage of culled fruit) confirms the relevance of developing methodologies that can incorporate non-linearity (Ober et al., 2015; Gezan et al., 2017). This purpose can be attained by different approaches. The simplest one is to incorporate a general matrix into the linear models made up of dummy variables. This model contains as many degrees of freedom as ploidy level per locus and allowing for any interaction structure between alleles (Enciso-Rodriguez et al., 2018; Amadeu et al., 2019). RKHS models (Gianola et al., 2006; Gianola et al., 2008; de los Campos et al., 2009) are also able to capture complex interaction patterns in a relatively straightforward manner. Alternatively, a CNN can be implemented using simply the raw data. Our analyses suggest that DL can perform better than additive and RKHS models for traits where the epistatic component is important and where narrow-sense heritability is low (e.g. percentage of culled fruit, **Figure 6**). The simulation study performed in this work (**Table 2**) suggested that BRR including additivity, dominance and the general dummy matrix described above can improve upon CNNs when the non-additive component is important, although CNNs were better than strict additive linear models. Additional analyses with a wider range of phenotypic traits, genetic structures and in larger datasets are needed to validate our results.

An underlying goal of our work was to investigate the effect of accounting for allele dosage in a GP context. Owing to the complex nature of polyploids, genotype calling can be a challenge and “diploidization”, i.e., considering a polyploid genome as diploid is usual (Bourke et al., 2018). Some studies have recently investigated the effect of accounting the ploidy level in prediction accuracy in polyploids (Endelman et al., 2018; Nyine et al., 2018; Amadeu et al., 2019; Lara et al., 2019; Zingaretti et al., 2019). As in these previous results (de Bem Oliveira et al., 2019; Zingaretti et al., 2019), here we found that “diploidization”, in which all heterozygous genotypes are pooled, is as efficient and accurate as polyploid genotyping for prediction purposes, albeit it is trait dependent. Therefore, we conjecture that genomic selection, particularly for low levels of ploidy, can pay off in polyploids even with simplified genotyping strategies. We need to be careful though as this approach may not be equally appropriate for all levels of ploidy and heterozygosity. For instance, this might be an issue with sugarcane (with ploidy starting from 2n=20) as most individuals will be heterozygous.

It is traditionally thought that DL requires extremely large datasets to be trained effectively (Alipanahi et al., 2015; Liang and Hu, 2015; Xiong et al., 2015). However, this and related works (Ma et al., 2017; Bellot et al., 2018; Montesinos-López et al., 2018a; Montesinos-López et al., 2019b) have shown that DL performance in GP is comparable to those of linear methods. Furthermore, the largest dataset analyzed so far with DL for prediction (~100k individuals) did not show a consistent advantage of DL (Bellot et al., 2018). Therefore, it seems that is the trait what really influences the success of DL and it appears not so critical the size of the dataset. This does not preclude, of course, that a large N is needed to advance in our knowledge on best GP strategies. In fact, a larger N can be especially recommended in clonally propagated species. It is well known that an efficient breeding program tests a low number of crosses with a high number of genotypes in each of them. A cross would need to be tested if not much information is available though. Numerous clonally propagated species of agricultural interest are polyploids, leading to high heterozygosity, non-linear interactions, and scarce prior knowledge about the crosses. In this scenario, as many cross-combinations as feasible should be produced to ensure the discovery and evaluation of the best genotypes (Grüneberg et al., 2009). The actual balance will depend on the level of epistasis and dominance. If dominance is large, then the best clone would be within families with good performance; if dominance is low, this is not necessarily so.

A drawback of DL models is that they lack biological or process interpretability and neither feature selection nor feature importance are obvious. In our opinion, GP algorithms are not too useful for providing biological insight into the genetic basis of phenotypes; genome wide association studies should be more appropriate. In all, our results suggest that DL performance improve as non-additive variance increases, a situation is usually encountered in fitness related traits.

DL hyperparameter tuning is critical and difficult, especially in terms of computational resources. Our analysis allows us to provide some generic recommendations though. First, we and others (Bellot et al., 2018; Montesinos-López et al., 2018a; Montesinos-López et al., 2019b) concluded that the predictive accuracy is mainly dependent of the trait, i.e., the architecture needs to be tuned for each trait individually. Second, here we show that the popular relu activation function is not necessarily a universally valid activation function, that interactions between hyperparameter combinations should be expected and that the number of convolutional filters and regularization in the first layers can have an important effect into the model performance (**Figure 5**). In general, we and other authors (Bellot et al., 2018; Waldmann, 2018) have reported that a shallow network is the best scenario in most cases. Nevertheless, DL can still be attractive because it does not require feature engineering, a critical step in most machine learning methods. A further strength of DL is its flexibility, e.g., it allows to define latent variables by using autoencoder or embedding as a generative latent variable model. In addition, networks, even if shallow, can model complex relationships employing any non-linear activation function.

Overall, there is no evidence that applying DL in GP applications necessarily improves the prediction accuracy upon that of classical linear model methods. PA depends on the trait and is affected by many factors; no one algorithm is uniformly better for all species and traits (Hu and Greene, 2018; Pérez-Enciso and Zingaretti, 2019). PA usually decays if heritability is low or in the presence of high epistatic effects. Even under these conditions though, Bayesian models were better than CNNs in almost all cases (**Tables S1**, **S2**, **Table 2**). Even if performance of DL for GP is not outstanding, we cannot ignore that plant breeding is based on both genotyping and phenotyping, and that high throughput phenotyping is critical for genomic dissection of complex traits (Cobb et al., 2013). Imaging and computer vision can be employed to measure the physiological, growth, development, and other phenotypic properties of plants with the advantage of being fast, non-invasive and a low-cost strategy (Fahlgren et al., 2015), hyperspectral imaging is useful to measure plant traits under say disease progression (Bergsträsser et al., 2015), infrared thermography is able to scan temperature and transpiration; NMR (nuclear magnetic resonance spectroscopy) and mass spectrometry (MS) are applied in plants metabolite evaluation (Hong et al., 2016). These examples should be an ideal scenario to neural networks as they involve imaging at high scale, complex, and heterogeneous datasets with multiple variables and outcome. In summary, we believe that the enormous amount of data that can be automatically recorded revolutionizing plant breeding and the flexible nature of neural networks makes them promising for meeting this future challenge.

## Data Availability Statement

The strawberry dataset analyzed for this study can be obtained from V.M. Whitaker (vwhitaker@ufl.edu); the blueberry dataset can be obtained from P.R. Muñoz (p.munoz@ufl.edu) or Dryad Digital Repository (accession number doi: 10.5061/dryad.kd4jq6h) and https://gsajournals.figshare.com/articles/Supplemental_Material_for_de_Bem_Oliveira_et_al_2019/7728365.

## Author Contributions

MP-E conceived and supervised research. VW, PM, LO, SG, and LF contributed experimental data. LZ developed software and performed research. LZ and MP-E wrote the initial manuscript draft. All authors contributed to discussion and to writing the final draft.

## Funding

LZ was supported by a PhD grant from the Ministry of Economy and Science (MINECO, Spain), by the MINECO grant AGL2016-78709-R to MP-E and from the EU through the BFU2016-77236-P (MINECO/AEI/FEDER, EU) and the “Centro de Excelencia Severo Ochoa 2016-2019” award SEV-2015-0533. VW and LO were supported by the US Department of Agriculture/National Institute of Food and Agriculture Specialty Crop Research Initiative (SCRI) project ‘RosBREED: Combining disease resistance with horticultural quality in new rosaceous cultivars' under Award Number 2014-51181-22378.

## Conflict of Interest

The authors declare that the research was conducted in the absence of any commercial or financial relationships that could be construed as a potential conflict of interest.

## References

[B1] Abadi M. Agarwal A. Barham P. Brevdo E. Chen Z. Citro C. (2015). TensorFlow: large-scale machine learning on heterogeneous systems. Software available tensorflow. Org. 1, 1–19. 10.1016/0076-6879(83)01039-3

[B2] Alipanahi B. Delong A. Weirauch M. T. Frey B. J. (2015). Predicting the sequence specificities of DNA- and RNA-binding proteins by deep learning. Nat. Biotechnol. 33, 831–838. 10.1038/nbt.3300 26213851

[B3] Amadeu R. R. Cellon C. Olmstead J. W. Garcia A. A. F. Resende M. F. R. Muñoz P. R. (2016). AGHmatrix: R package to construct relationship matrices for autotetraploid and diploid species: a blueberry example. Plant Genome 9. 10.3835/plantgenome2016.01.0009 27902800

[B4] Amadeu R. R. Ferrão L. F. V. Oliveira I. de B. Benevenuto J. Endelman J. B. (2019). Impact of dominance effects on autotetraploid genomic prediction. Crop Sci. 0, 0. 10.2135/cropsci2019.02.0138

[B5] Autonomio Talos [Computer software] (2019). Retrieved from http://github.com/autonomio/talos.

[B6] Azodi C. B. McCarren A. Roantree M. Campos G. de los Shiu S.-H. (2019). Benchmarking algorithms for genomic prediction of complex traits. bioRxiv 614479. 10.1101/614479 PMC6829122 31533955

[B7] Badrinarayanan V. Kendall A. Cipolla R. (2017). SegNet: A Deep Convolutional encoder-decoder architecture for image segmentation. IEEE Trans. Pattern Anal. Mach. Intell. 39, 2481–2495. 10.1109/TPAMI.2016.2644615 28060704

[B8] Bassil N. V. Davis T. M. Zhang H. Ficklin S. Mittmann M. Webster T. (2015). Development and preliminary evaluation of a 90 K Axiom® SNP array for the allo-octoploid cultivated strawberry Fragaria × ananassa. BMC Genomics 16, 155. 10.1186/s12864-015-1310-1 25886969 PMC4374422

[B9] Bawa V. S. Kumar V. (2019). Linearized sigmoidal activation: a novel activation function with tractable non-linear characteristics to boost representation capability. Expert Syst. Appl. 120, 346–356. 10.1016/j.eswa.2018.11.042

[B10] Bellot P. de Los Campos G. Pérez-Enciso M. (2018). Can deep learning improve genomic prediction of complex human traits? Genetics 210, 809–819. 10.1534/genetics.118.301298 30171033 PMC6218236

[B11] Benevenuto J. Ferrão L. F. V. Amadeu R. R. Munoz P. (2019). How can a high-quality genome assembly help plant breeders? Gigascience 8, 1–4. 10.1093/gigascience/giz068 PMC6558523 31184361

[B12] Bergsträsser S. Fanourakis D. Schmittgen S. Cendrero-Mateo M. Jansen M. Scharr H. (2015). HyperART: non-invasive quantification of leaf traits using hyperspectral absorption-reflectance-transmittance imaging. Plant Methods 11, 1. 10.1186/s13007-015-0043-0 25649124 PMC4302522

[B13] Bernardo R. (2008). Molecular markers and selection for complex traits in plants: Learning from the last 20 years. Crop Sci. 48, 1649–1664. 10.2135/cropsci2008.03.0131

[B14] Bourke P. M. Voorrips R. E. Visser R. G. F. Maliepaard C. (2018). Tools for genetic studies in experimental populations of polyploids. Front. Plant Sci. 9, 513. 10.3389/fpls.2018.00513 29720992 PMC5915555

[B15] Castillo-Juárez H. Campos-Montes G. R. Caballero-Zamora A. Montaldo H. H. (2015). Genetic improvement of pacific white shrimp [Penaeus (Litopenaeus) vannamei]: perspectives for genomic selection. Front. Genet. 6, 93. 10.3389/fgene.2015.00093 25852740 PMC4371756

[B16] Chan M. Scarafoni D. Duarte R. Thornton J. Skelly L. (2018). “Learning network architectures of deep CNNs under resource constraints,” in IEEE Computer Society Conference on Computer Vision and Pattern Recognition Workshops. (IEEE), 1784–1791. 10.1109/CVPRW.2018.00222

[B17] Cho M. Hegde C. (2019). “Reducing the Search Space for Hyperparameter Optimization Using Group Sparsity,” in ICASSP, IEEE International Conference on Acoustics, Speech and Signal Processing - Proceedings (New York City: Institute of Electrical and Electronics Engineers Inc.), 3627–3631. 10.1109/ICASSP.2019.8682434

[B18] Chollet F. (2015). Keras: deep learning library for theano and tensorflow 7, T1 Available at: url: https://keras.io/k.

[B19] Cobb J. N. DeClerck G. Greenberg A. Clark R. McCouch S. (2013). Next-generation phenotyping: requirements and strategies for enhancing our understanding of genotype–phenotype relationships and its relevance to crop improvement. Theor. Appl. Genet. 126, 867–887. 10.1007/s00122-013-2066-0 23471459 PMC3607725

[B20] Colle M. Leisner C. P. Wai C. M. Ou S. Bird K. A. Wang J. (2019). Haplotype-phased genome and evolution of phytonutrient pathways of tetraploid blueberry. Gigascience 8, 3. 10.1093/gigascience/giz012 PMC6423372 30715294

[B21] Crossa J. J. Beyene Y. Kassa S. Pérez P. Hickey J. M. Chen C. (2013a). Genomic prediction in maize breeding populations with genotyping-by-sequencing. *G3*. (Bethesda) 3, 1903–1926. 10.1534/g3.113.008227 PMC3815055 24022750

[B22] Crossa J. Pérez P. Hickey J. Burgueño J. Ornella L. Ceró N-Rojas J. (2013b). Genomic prediction in CIMMYT maize and wheat breeding programs. Heredity (Edinb). 112, 48–60. 10.1038/hdy.2013.16 23572121 PMC3860161

[B23] Daetwyler H. D. Calus M. P. L. Pong-Wong R. de los Campos G. Hickey J. M. (2013). Genomic prediction in animals and plants: simulation of data, validation, reporting, and benchmarking. Genetics 193, 347–365. 10.1534/genetics.112.147983 23222650 PMC3567728

[B24] de Bem Oliveira I. Resende M. F. R. Ferrão L. F. V. Amadeu R. R. Endelman J. B. Kirst M. (2019). Genomic prediction of autotetraploids; influence of relationship matrices, allele dosage, and continuous genotyping calls in phenotype prediction. *G3*. Genes Genomes Genet. 9, g3.400059.2019. 10.1534/g3.119.400059 PMC6469427 30782769

[B25] de los Campos G. Gianola D. Rosa G. J. M. (2009). Reproducing kernel Hilbert spaces regression: a general framework for genetic evaluation1. J. Anim. Sci. 87, 1883–1887. 10.2527/jas.2008-1259 19213705

[B26] De Los Campos G. Naya H. Gianola D. Crossa J. Legarra A. Manfredi E. (2009). Predicting quantitative traits with regression models for dense molecular markers and pedigree. Genetics 182, 375–385. 10.1534/genetics.109.101501 19293140 PMC2674834

[B27] de Los Campos G. Gianola D. Rosa G. J. Weigel K. A. Crossa J. (2010). Semi-parametric genomic-enabled prediction of genetic values using reproducing kernel Hilbert spaces methods. Genet. Res. (Camb). 92, 295–308. 10.1017/S0016672310000285 20943010

[B28] de los Campos G. Hickey J. M. Pong-Wong R. Daetwyler H. D. Calus M. P. L. (2013). Whole-genome regression and prediction methods applied to plant and animal breeding. Genetics 193, 327–345. 10.1534/genetics.112.143313 22745228 PMC3567727

[B29] Deng L. Liu Y. (2018). Deep Learning in Natural Language. (Springer) 10.1007/978-981-10-5209-5_11

[B30] Drayer B. Brox T. (2014). “Training deformable object models for human detection based on alignment and clustering,” in Lecture Notes in Computer Science (including subseries Lecture Notes in Artificial Intelligence and Lecture Notes in Bioinformatics). (Cham, Springer), 406–420. 10.1007/978-3-319-10602-1_27

[B31] Duangjit J. Causse M. Sauvage C. (2016). Efficiency of genomic selection for tomato fruit quality. Mol. Breed. 36, 29. 10.1007/s11032-016-0453-3

[B32] Enciso-Rodriguez F. Douches D. Lopez-Cruz M. Coombs J. de los Campos G. (2018). Genomic selection for late blight and common scab resistance in tetraploid potato (Solanum tuberosum). *G3*. Genes Genomes Genet. 8, 2471–2481. 10.1534/g3.118.200273 PMC6027896 29794167

[B33] Endelman J. B. Carley C. A. S. Bethke P. C. Coombs J. J. Clough M. E. da Silva W. L. (2018). Genetic variance partitioning and Genome-Wide prediction with allele dosage information in autotetraploid Potato. Genetics 209, 77–87. 10.1534/genetics.118.300685 29514860 PMC5937173

[B34] Fahlgren N. Gehan M. A. Baxter I. (2015). Lights, camera, action: high-throughput plant phenotyping is ready for a close-up. Curr. Opin. Plant Biol. 24, 93–99. 10.1016/j.pbi.2015.02.006 25733069

[B35] Feng J. Lu S. (2019). “Performance Analysis of Various Activation Functions in Artificial Neural Networks,” in Journal of Physics: Conference Series. (IOP). 10.1088/1742-6596/1237/2/022030

[B36] Ferrão L. F. V. Benevenuto J. Oliveira I. de B. Cellon C. Olmstead J. (2018). Insights into the genetic basis of blueberry fruit-related traits using diploid and polyploid models in a GWAS context. Front. Ecol. Evol. 6, 107. 10.3389/fevo.2018.00107

[B37] Gallais A. (2003). Quantitative genetics and breeding methods in autopolyploids plants. (Quae).

[B38] Gapare W. Liu S. Conaty W. Zhu Q. H. Gillespie V. Llewellyn D. (2018). Historical datasets support genomic selection models for the prediction of cotton fiber quality phenotypes across multiple environments. *G3*. Genes Genomes Genet. 8, 1721–1732. 10.1534/g3.118.200140 PMC5940163 29559536

[B39] Gerard D. Ferrão L. F. V. Garcia A. A. F. Stephens M. (2018). Genotyping polyploids from messy sequencing data. Genetics 210, 789–807. 10.1534/genetics.118.301468 30185430 PMC6218231

[B40] Gezan S. A. Osorio L. F. Verma S. Whitaker V. M. (2017). An experimental validation of genomic selection in octoploid strawberry. Hortic. Res. 4, 16070. 10.1038/hortres.2016.70 28090334 PMC5225750

[B41] Gianola D. Fernando R. L. Stella A. (2006). Genomic-assisted prediction of genetic value with semiparametric procedures. Genetics 173, 1761–1776. 10.1534/genetics.105.049510 16648593 PMC1526664

[B42] Gianola D. van Kaam J. B. C. H. M. Toro M. A. (2008). Reproducing kernel hilbert spaces regression methods for genomic assisted prediction of quantitative traits. Genetics 178, 2289–2303. 10.1534/genetics.107.084285 18430950 PMC2323816

[B43] Gianola D. (2013). Priors in whole-genome regression: the bayesian alphabet returns. Genetics 194, 573–596. 10.1534/genetics.113.151753 23636739 PMC3697965

[B44] González-Camacho J. M. de los Campos G. Pérez P. Gianola D. Cairns J. E. Mahuku G. (2012). Genome-enabled prediction of genetic values using radial basis function neural networks. Theor. Appl. Genet. 125, 759–771. 10.1007/s00122-012-1868-9 22566067 PMC3405257

[B45] González-Recio O. Rosa G. J. M. Gianola D. (2014). Machine learning methods and predictive ability metrics for genome-wide prediction of complex traits. Livest. Sci. 166, 217–231. 10.1016/j.livsci.2014.05.036

[B46] Goodfellow I. Bengio Y. Courville A. (2016). Deep Learning (Cambridge: MIT Press Cambridge).

[B47] Grüneberg W. Mwanga R. Andrade M. Espinoza J. (2009). Selection methods. Part 5: breeding clonally propagated crops. Plant Breed. Farmer Particip. 275–322. Available at: http://www.cabdirect.org/abstracts/20103075062.html.

[B48] Hancock J. F. Sjulin T. M. Lobos G. A. (2008). “Strawberries,” in Temperate fruit crop breeding, vol. 393–437 (Wallingford, UK: Springer, Dordrecht). 10.1007/978-1-4020-6907-9

[B49] Henderson C. R. (1984). Best linear unbiased prediction of performance and breeding value. Biometrics, 172–192. Available at papers3//publication/uuid/627506AA-ACB7-491A-B468-9A3B5C2A52EC. 10.2527/jas1985.601111x

[B50] Hong J. Yang L. Zhang D. Shi J. (2016). Plant metabolomics: An indispensable system biology tool for plant science. Int. J. Mol. Sci. 17, 767. 10.3390/ijms17060767 PMC4926328 27258266

[B51] Hornik K. Stinchcombe M. White H. (1990). Universal approximation of an unknown mapping and its derivatives using multilayer feedforward networks. Neural Networks 3, 551–560. 10.1016/0893-6080(90)90005-6

[B52] Hu Q. Greene C. S. (2018). Parameter tuning is a key part of dimensionality reduction *via* deep variational autoencoders for single cell RNA transcriptomics. bioRxiv 385534. 10.1101/385534 PMC6417816 30963075

[B53] Işin A. Direkoǧlu C. Şah M. (2016). “Review of MRI-based Brain Tumor Image Segmentation Using Deep Learning Methods,” in Procedia Computer Science (Amsterdam: Elsevier), 317–324. 10.1016/j.procs.2016.09.407

[B54] Juliana P. Poland J. Huerta-Espino J. Shrestha S. Crossa J. Crespo-Herrera L. (2019). Improving grain yield, stress resilience and quality of bread wheat using large-scale genomics. Nat. Genet. 51, 1530–1539. 10.1038/s41588-019-0496-6 31548720

[B55] Lara L. A. D. C. Santos M. F. Jank L. Chiari L. De Vilela M. M. Amadeu R. R. (2019). Genomic selection with allele dosage in Panicum maximum Jacq. *G3*. Genes Genomes Genet. 9, 2463–2475. 10.1534/g3.118.200986 PMC6686918 31171567

[B56] Le N. Q. K. Huynh T. T. Yapp E. K. Y. Yeh H. Y. (2019). Identification of clathrin proteins by incorporating hyperparameter optimization in deep learning and PSSM profiles. Comput. Methods Prog. Biomed. 177, 81–88. 10.1016/j.cmpb.2019.05.016 31319963

[B57] LeCun Y. Bengio Y. Hinton G. (2015). Deep learning. Nature 521, 436–444. 10.1038/nature14539 26017442

[B58] Liang M. Hu X. (2015). “Recurrent convolutional neural network for object recognition,” in Proceedings of the IEEE Computer Society Conference on Computer Vision and Pattern Recognition. (IEEE) 3367–3375. 10.1109/CVPR.2015.7298958

[B59] Liaw A. Wiener M. (2002). Classification and regression by random forest. R News 2, 18–22.

[B60] Ma W. Qiu Z. Song J. Cheng Q. Ma C. (2017). DeepGS: predicting phenotypes from genotypes using deep learning. bioRxiv 241414. 10.1101/241414

[B61] Maas A. L. Hannun A. Y. Ng A. Y. (2013). “Rectifier nonlinearities improve neural network acoustic models,” in in ICML Workshop on Deep Learning for Audio, Speech and Language Processing. Available at: https://www.semanticscholar.org/paper/Rectifier-Nonlinearities-Improve-Neural-Network-Maas/367f2c63a6f6a10b3b64b8729d601e69337ee3cc [Accessed October 11, 2019].

[B62] MacKay T. F. (2009). Q & A: genetic analysis of quantitative traits. J. Biol. 8, 23. 10.1186/jbiol133 19435484 PMC2689437

[B63] Meuwissen T. H. E. Hayes B. J. Goddard M. E. (2001). Prediction of total genetic value using genome-wide dense marker maps. Genetics 157, 1819–1829. 11290733 10.1093/genetics/157.4.1819 PMC1461589

[B64] Meuwissen T. Hayes B. Goddard M. (2013). Accelerating improvement of livestock with genomic selection. Annu. Rev. Anim. Biosci. 1, 221–237. 10.1146/annurev-animal-031412-103705 25387018

[B65] Min S. Lee B. Yoon S. (2017). Deep learning in bioinformatics. Brief. Bioinform. 18, 851–869. 10.1093/bib/bbw068 27473064

[B66] Montesinos-López A. Montesinos-López O. A. Hernández-Suárez C. M. Gianola D. Crossa J. (2018a). Multi-environment genomic prediction of plant traits using deep learners with dense architecture. *G3*. Genes Genomes Genet. 8 (12), 3813–3828. 10.1534/g3.118.200740 PMC6288841 30291107

[B67] Montesinos-López O. A. Montesinos-López A. Crossa J. Gianola D. Hernández-Suárez C. M. Martín-Vallejo J. (2018b). Multi-trait, multi-environment deep learning modeling for genomic-enabled prediction of plant traits. *G3*. Genes Genomes Genet. 8, 3829–3840. 10.1534/g3.118.200728 PMC6288830 30291108

[B68] Montesinos-López O. A. Martín-Vallejo J. Crossa J. Gianola D. Hernández-Suárez C. M. Montesinos-López A. (2019a). A benchmarking between deep learning, support vector machine and Bayesian threshold best linear unbiased prediction for predicting ordinal traits in plant breeding. *G3*. Genes Genomes Genet. 9, 601–618. 10.1534/g3.118.200998 PMC6385991 30593512

[B69] Montesinos-López O. A. Martín-Vallejo J. Crossa J. Gianola D. Hernández-Suárez C. M. Montesinos-López A. (2019b). New deep learning genomic-based prediction model for multiple traits with binary, ordinal, and continuous phenotypes. *G3*. Genes Genomes Genet. 9, 1545–1556. 10.1534/g3.119.300585 PMC6505163 30858235

[B70] Namin S. T. Esmaeilzadeh M. Najafi M. Brown T. B. Borevitz J. O. (2018). Deep phenotyping: deep learning for temporal phenotype/genotype classification. Plant Methods 14, 66. 10.1186/s13007-018-0333-4 30087695 PMC6076396

[B71] Nyine M. Uwimana B. Blavet N. Hřibová E. Vanrespaille H. Batte M. (2018). Genomic prediction in a multiploid crop: genotype by environment interaction and allele dosage effects on predictive ability in Banana. Plant Genome 11, 1–16. 10.3835/plantgenome2017.10.0090 PMC12962448 30025016

[B72] Ober U. Huang W. Magwire M. Schlather M. Simianer H. Mackay T. F. C. (2015). Accounting for genetic architecture improves sequence based genomic prediction for a drosophila fitness trait. PloS One 10, e0126880. 10.1371/journal.pone.0126880 25950439 PMC4423967

[B73] Osborn T. C. Chris Pires J. Birchler J. A. Auger D. L. Chen Z. J. Lee H. S. (2003). Understanding mechanisms of novel gene expression in polyploids. Trends Genet. 19, 141–147. 10.1016/S0168-9525(03)00015-5 12615008

[B74] Pérez P. De Los Campos G. (2014). Genome-wide regression and prediction with the BGLR statistical package. Genetics 198, 483–495. 10.1534/genetics.114.164442 25009151 PMC4196607

[B75] Pérez-Enciso Zingaretti (2019). A guide on deep learning for complex trait genomic prediction. Genes (Basel). 10, 553. 10.3390/genes10070553 PMC6678200 31330861

[B76] Pattanayak S. (2017). “Unsupervised Learning with Restricted Boltzmann Machines and Auto-encoders,” in Pro Deep Learning with TensorFlow (Berkeley, CA: Apress), 279–343. 10.1007/978-1-4842-3096-1_5

[B77] Pouladi F. Salehinejad H. Gilani A. M. (2016). Deep recurrent neural networks for sequential phenotype prediction in genomics. arXiv Prepr. arXiv. *1511**02554* Available at: https://arxiv.org/pdf/1511.02554.pdf [Accessed March 4, 2019].

[B78] Rajaraman S. Jaeger S. Antani S. K. (2019). Performance evaluation of deep neural ensembles toward malaria parasite detection in thin-blood smear images. PeerJ 7, e6977. 10.7717/peerj.6977 31179181 PMC6544011

[B79] Santantonio N. Jannink J. L. Sorrells M. (2019). A low resolution epistasis mapping approach to identify chromosome arm interactions in allohexaploid wheat. *G3*. Genes Genomes Genet. 9, 675–684. 10.1534/g3.118.200646 PMC6404624 30455184

[B80] Slater A. T. Cogan N. O. I. Forster J. W. Hayes B. J. Daetwyler H. D. (2016). Improving genetic gain with genomic selection in autotetraploid potato. Plant Genome 9, 1–15. 10.3835/plantgenome2016.02.0021 27902807

[B81] Tibshirani R. (1996). Regression shrinkage and selection via the Lasso. J. R. Stat. Soc. Ser. B 58, 267–288. 10.1111/j.2517-6161.1996.tb02080.x

[B82] VanRaden P. M. (2008). Efficient methods to compute genomic predictions. J. Dairy Sci. 91, 4414–4423. 10.3168/jds.2007-0980 18946147

[B83] Vitezica Z. G. Varona L. Legarra A. (2013). On the additive and dominant variance and covariance of individuals within the genomic selection scope. Genetics 195, 1223–1230. 10.1534/genetics.113.155176 24121775 PMC3832268

[B84] Waldmann P. (2018). Approximate bayesian neural networks in genomic prediction. Genet. Sel. Evol. 50, 70. 10.1186/s12711-018-0439-1 30577737 PMC6303864

[B85] Widder D. V. (1954). The convolution transform. Bull. Am. Math. Soc. 60, 444–456. 10.1090/S0002-9904-1954-09828-2

[B86] Wiggans G. R. Cole J. B. Hubbard S. M. Sonstegard T. S. (2017). Genomic Selection in dairy cattle: the USDA experience. Annu. Rev. Anim. Biosci. 5, 309–327. 10.1146/annurev-animal-021815-111422 27860491

[B87] Xiong H. Y. Alipanahi B. Lee L. J. Bretschneider H. Merico D. Yuen R. K. C. (2015). RNA splicing. The human splicing code reveals new insights into the genetic determinants of disease. Science 347, 1254806. 10.1126/science.1254806 25525159 PMC4362528

[B88] Yoo Y. J. (2019). Hyperparameter optimization of deep neural network using univariate dynamic encoding algorithm for searches. Knowledge-Based Syst. 178, 74–83. 10.1016/j.knosys.2019.04.019

[B89] Young S. R. Rose D. C. Karnowski T. P. Lim S.-H. Patton R. M. (2015). “Optimizing deep learning hyper-parameters through an evolutionary algorithm. Proceedings of the Workshop on Machine Learning in High-Performance Computing Environments - MLHPC ‘15,” (New York, New York, USA: ACM Press), 1–5. 10.1145/2834892.2834896

[B90] Zingaretti M. L. Monfort A. Pérez-Enciso M. (2019). pSBVB: a versatile simulation tool to evaluate genomic selection in polyploid species. *G3*. (Bethesda) 9, 327–334. 10.1534/g3.118.200942 PMC6385978 30573468

