# Supplementary material for: Exploring Deep Learning for Complex Trait Genomic Prediction in Polyploid Outcrossing Species
Source: Front Plant Sci. 2020 Feb 6;11:25. doi: 10.3389/fpls.2020.00025 (PMC7015897; doi:10.3389/fpls.2020.00025)

*Supplementary Material*

# Exploring deep learning for complex trait genomic prediction in polyploid outcrossing species

**L.M. Zingaretti[1*], S.A. Gezan[2], L.F. Ferrão[3], L.F. Osorio[4], A. Monfort[1,5], P.R. Muñoz[3], V.M. Whitaker[4], M. Pérez-Enciso[1,6*]**

[1] Centre for Research in Agricultural Genomics (CRAG), CSIC-IRTA-UAB-UB Consortium, 08193 Bellaterra, Barcelona, Spain

[2] School of Forest Resources and Conservation, University of Florida, 363 Newins-Ziegler Hall, PO Box 110410, Gainesville, FL 32611-0410, USA

[3] Blueberry Breeding and Genomics Lab, Horticultural Sciences Department, University of Florida, Gainesville, FL 32611, USA

[4] IFAS Gulf Coast Research and Education Center, University of Florida, 14625 CR 672, Wimauma, FL 33598, USA

[5] Institut de Recerca i Tecnologia Agroalimentàries (IRTA), 08193 Barcelona, Spain

[6] ICREA, Passeig de Lluís Companys 23, 08010 Barcelona, Spain

*** Correspondence:**
Corresponding authors:
laura.zingaretti@cragenomica.es

miguel.perez@uab.es

**Figure S1:** a) Principal component (PC) representation of the strawberry genotypes, each season (T2, T4, T6, T8 and T10) is in a different color; b) Principal component representation of the blueberry genotypes, training and validation individuals are colored differently.

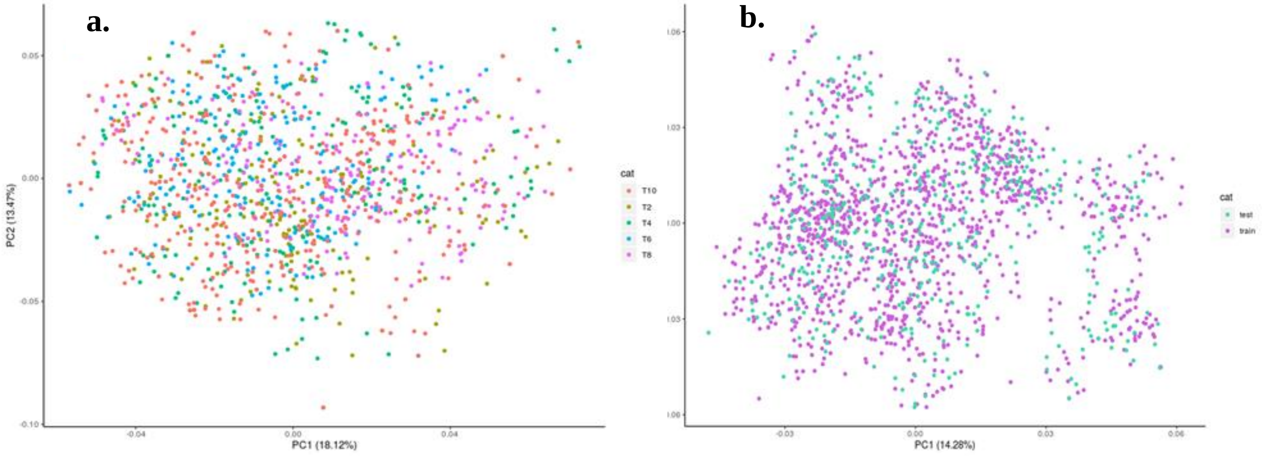

Supplement: Supplementary file 1 [file Image_1.pdf]
