# Supplementary material for: Exploring Deep Learning for Complex Trait Genomic Prediction in Polyploid Outcrossing Species
Source: Front Plant Sci. 2020 Feb 6;11:25. doi: 10.3389/fpls.2020.00025 (PMC7015897; doi:10.3389/fpls.2020.00025)
Supplement: Supplementary file 2 [file Table_1.docx]

*Supplementary Material*

Exploring deep learning for complex trait genomic prediction in polyploid outcrossing species

**L.M. Zingaretti^1*^, S.A. Gezan^2^, L.F. Ferrão^3^, L.F. Osorio^4^, A. Monfort^1,5^, P.R. Muñoz^3^, V.M. Whitaker^4^, M. Pérez-Enciso^1,6*^**

^1^ Centre for Research in Agricultural Genomics (CRAG), CSIC-IRTA-UAB-UB Consortium, 08193 Bellaterra, Barcelona, Spain

^2^ School of Forest Resources and Conservation, University of Florida, 363 Newins-Ziegler Hall, PO Box 110410, Gainesville, FL 32611-0410, USA

^3^ Blueberry Breeding and Genomics Lab, Horticultural Sciences Department, University of Florida, Gainesville, FL 32611, USA

^4^ IFAS Gulf Coast Research and Education Center, University of Florida, 14625 CR 672, Wimauma, FL 33598, USA

^5^ Institut de Recerca i Tecnologia Agroalimentàries (IRTA), 08193 Barcelona, Spain

^6^ ICREA, Passeig de Lluís Companys 23, 08010 Barcelona, Spain

*** Correspondence:**Corresponding authors:
[laura.zingaretti@cragenomica.es](mailto:laura.zingaretti@cragenomica.es)

[miguel.perez@uab.es](mailto:miguel.perez@uab.es)

**Table S1:** Optimum hyperparameter choice in strawberry data.

| Trait | Activation function CNN / MLP | # CNN Layers /  # Filters | # MLP layers /  # Neurons | Weight decay | Dropout rate CNN / MLP |
| --- | --- | --- | --- | --- | --- |
| Brix | Linear / relu | 1 / 128 | 3 / 12 | 0.001 | 0.1 / 0.0 |
| MktWtE | Linear / linear | 1 / 128 | 10 / 4 | 0.001 | 0.1 / 0.0 |
| MktWtT | Linear / linear | 1 / 128 | 1 / 32 | 0.001 | 0.2 / 0.001 |
| AveWtT | Linear / linear | 1 / 128 | 10 / 8 | 0.001 | 0.2 / 0.01 |
| CulsTPer | Linear / relu | 1 / 128 | 1 / 128 | 0.01 | 0.01 / 0.0 |

| Learning rate was 0.0025 except for CulsTPer (lr = 0.01). |
| --- |
