# Supplementary material for: Exploring Deep Learning for Complex Trait Genomic Prediction in Polyploid Outcrossing Species
Source: Front Plant Sci. 2020 Feb 6;11:25. doi: 10.3389/fpls.2020.00025 (PMC7015897; doi:10.3389/fpls.2020.00025)
Supplement: Supplementary file 4 [file Table_3.docx]

*Supplementary Material*

Exploring deep learning for complex trait genomic prediction in polyploid outcrossing species

**L.M. Zingaretti^1*^, S.A. Gezan^2^, L.F. Ferrão^3^, L.F. Osorio^4^, A. Monfort^1,5^, P.R. Muñoz^3^, V.M. Whitaker^4^, M. Pérez-Enciso^1,6*^**

^1^ Centre for Research in Agricultural Genomics (CRAG), CSIC-IRTA-UAB-UB Consortium, 08193 Bellaterra, Barcelona, Spain

^2^ School of Forest Resources and Conservation, University of Florida, 363 Newins-Ziegler Hall, PO Box 110410, Gainesville, FL 32611-0410, USA

^3^ Blueberry Breeding and Genomics Lab, Horticultural Sciences Department, University of Florida, Gainesville, FL 32611, USA

^4^ IFAS Gulf Coast Research and Education Center, University of Florida, 14625 CR 672, Wimauma, FL 33598, USA

^5^ Institut de Recerca i Tecnologia Agroalimentàries (IRTA), 08193 Barcelona, Spain

^6^ ICREA, Passeig de Lluís Companys 23, 08010 Barcelona, Spain

*** Correspondence:**Corresponding authors:
[laura.zingaretti@cragenomica.es](mailto:laura.zingaretti@cragenomica.es)

[miguel.perez@uab.es](mailto:miguel.perez@uab.es)

**Table S3:** CNN architecture to evaluate the predictive ability for each of the 15 simulated models in Table 2.

| **Replicate** | **Architecture** | **Activation function (fully connected layer)** | **Nº of convolutions layers (Nº of Filters)** | **Nº of fully connected layers (Nº of neurons)** | **Weight decay [Fully connected layer]**  **(Output layer)** | **Dropout -CNN layer (Fully connected layer)** |
| --- | --- | --- | --- | --- | --- | --- |
| **1** | Additive | relu (tanh) | 1 (32) | 1 (12) | 0.001[0.001] (0) | 0 (0.1) |
| **2** | Additive | relu (tanh) | 1 (32) | 1 (12) | 0.001[0.001] (0) | 0 (0.1) |
| **3** | Additive | Linear(linear) | 1 (128) | 1 (12) | 0.001[0.001] (0) | 0 (0.1) |
| **4** | Additive | Linear(linear) | 1 (32) | 1 (8) | 0.001[0.001] (0) | 0 (0) |
| **5** | Additive | Linear(linear) | 1 (32) | 1 (8) | 0.001[0.001] (0) | 0 (0) |
| **6** | Mixed | Linear(linear) | 1 (32) | 1 (8) | 0.001[0.001] (0) | 0 (0) |
| **7** | Mixed | relu(tanh) | 1 (64) | 1 (8) | 0.001[0.001] (0) | 0 (0) |
| **8** | Mixed | relu(tanh) | 1 (64) | 1 (12) | 0.001[0.001] (0) | 0 (0.01) |
| **9** | Mixed | tanh(tanh) | 1 (64) | 2 (8) | 0.001[0.001] (0) | 0 (0) |
| **10** | Mixed | Tanh(linear) | 1 (256) | 1 (10) | 0.001[0.001] (0) | 0 (0.2) |
| **11** | Epistatic | relu(linear) | 1 (32) | 1 (8) | 0.001[0.001] (0) | 0 (0) |
| **12** | Epistatic | Tanh (linear) | 1 (256) | 1 (10) | 0.01[0.001] (0) | 0 (0.2) |
| **13** | Epistatic | relu (linear) | 1 (128) | 1 (10) | 0.001[0.001] (0) | 0 (0) |
| **14** | Epistatic | relu (tanh) | 1 (128) | 1 (10) | 0.001[0] (0) | 0 (0) |
| **15** | Epistatic | tanh (linear) | 1 (128) | 1 (8) | 0.001[0.001] (0) | 0 (0.05) |
